# *In Situ* Assessment of Porcine Osteochondral Repair Tissue in the Visible–Near Infrared Spectral Region

**DOI:** 10.3389/fbioe.2022.885369

**Published:** 2022-08-23

**Authors:** Shital Kandel, William Querido, Jessica M. Falcon, Hannah M. Zlotnick, Ryan C. Locke, Brendan Stoeckl, Jay M. Patel, Chetan A. Patil, Robert L. Mauck, Nancy Pleshko

**Affiliations:** ^1^ Department of Bioengineering, Temple University, Philadelphia, PA, United States; ^2^ Department of Bioengineering, University of Pennsylvania, Philadelphia, PA, United States; ^3^ Department of Orthopedics, Emory University, Atlanta, GA, United States

**Keywords:** visible–near infrared (Vis-NIR), fiber optic spectroscopy, cartilage repair, microfracture, *in situ* optical spectroscopy

## Abstract

Standard assessment of cartilage repair progression by visual arthroscopy can be subjective and may result in suboptimal evaluation. Visible–near infrared (Vis-NIR) fiber optic spectroscopy of joint tissues, including articular cartilage and subchondral bone, provides an objective approach for quantitative assessment of tissue composition. Here, we applied this technique in the 350–2,500 nm spectral region to identify spectral markers of osteochondral tissue during repair with the overarching goal of developing a new approach to monitor repair of cartilage defects *in vivo*. Full thickness chondral defects were created in Yucatan minipigs using a 5-mm biopsy punch, and microfracture (MFx) was performed as a standard technique to facilitate repair. Tissues were evaluated at 1 month (in adult pigs) and 3 months (in juvenile pigs) post-surgery by spectroscopy and histology. After euthanasia, Vis-NIR spectra were collected *in situ* from the defect region. Additional spectroscopy experiments were carried out *in vitro* to aid in spectral interpretation. Osteochondral tissues were dissected from the joint and evaluated using the conventional International Cartilage Repair Society (ICRS) II histological scoring system, which showed lower scores for the 1-month than the 3-month repair tissues. In the visible spectral region, hemoglobin absorbances at 540 and 570 nm were significantly higher in spectra from 1-month repair tissue than 3-month repair tissue, indicating a reduction of blood in the more mature repair tissue. In the NIR region, we observed qualitative differences between the two groups in spectra taken from the defect, but differences did not reach significance. Furthermore, spectral data also indicated that the hydrated environment of the joint tissue may interfere with evaluation of tissue water absorbances in the NIR region. Together, these data provide support for further investigation of the visible spectral region for assessment of longitudinal repair of cartilage defects, which would enable assessment during routine arthroscopy, particularly in a hydrated environment.

## Introduction

Articular cartilage, a connective tissue present at the end of long bones in diarthrodial joints, provides a smooth and lubricated surface for bone articulation without friction. It is composed of approximately 80 wt% water and 20% extracellular matrix, which consists of proteins including collagen type II and proteoglycans (Sophia Fox et al., 2009; Gao et al., 2014). Chondrocytes are the primary cells in this tissue that represent about 2% of the total volume of healthy adult cartilage. This tissue is also characterized by a lack of blood vessels, nerves, and lymphatics; thus, when an injury occurs, the tissue healing is very limited (Suh et al., 1995; Poole et al., 2001). Ultimately, progression of an injury or degradation of this tissue can result in the debilitating disease osteoarthritis (OA).

Osteoarthritis causes painful movement and discomfort and must be treated early for the best chance of success (Braun and Gold, 2012). Microfracture treatments are widely used for repair of chondral defects associated with OA, in particular for small defects less than ∼2 cm^2^ (Mithoefer et al., 2009). In this approach, a defect is repaired using a sharp tool (awl) to puncture holes in the subchondral plate, which allows blood and mesenchymal stem cells that reside in the bone marrow to migrate to the defect site and initiate repair (Steadman et al., 2010; Fisher et al., 2015). The repair process, however, frequently results in formation of undesirable fibrocartilage rich in collagen type I (Steadman et al., 2010; Kim et al., 2015; Vega et al., 2017).

Current gold-standard techniques for repair assessment include the use of the ICRS II scoring system, developed by the International Society of Cartilage Repair (ICRS) (Mainil-Varlet et al., 2010). This is a destructive approach based on the histology scoring of the repair tissue, and since this is an end-point test, longitudinal repair of the tissue cannot be assessed. Furthermore, second-look biopsies of repairing joint tissues are not the standard of care in the US or Europe (Brun et al., 2008). Similarly, another approach for assessment of tissue repair utilizes the Oswestry Arthroscopy Score. This scoring technique includes a visual and tactile assessment of repair tissue and can be performed arthroscopically (Paatela et al., 2020). However, the scoring can be subjective and may result in suboptimal assessment of defect repair (Brismar et al., 2002).

Infrared (IR) spectroscopic analysis has been used in evaluation of cartilage and bone tissue in both the mid-IR (MIR; 4,000–400 cm^−1^ or 2,500–25,000 nm) and near-IR (NIR; 10,000–4,000 cm^−1^ or 1000–2,500 nm) spectral regions (Querido et al., 2021). The NIR region has an advantage in the analysis of thick tissues as radiation can penetrate and be detected up to several millimeters into the tissue, including in cartilage (Padalkar and Pleshko, 2015). Considering that an average human articular cartilage ranges from 1 to 3 mm thickness, information from the entire cartilage depth can be obtained with NIR spectral analysis. NIR fiber optic spectroscopy has been previously used to non-destructively monitor matrix development in tissue-engineered cartilage constructs *in situ* (Hanifi et al., 2017; Yousefi et al., 2017; Kandel et al., 2020) and to assess proteoglycan content and cartilage thickness (Sarin et al., 2019). In particular, the baseline offset of NIR raw spectra have shown to be associated with an increase in the matrix development in engineered cartilage (Kandel et al., 2020). Additionally, the absorbances at 5,800 and 5,900 cm^−1^ have also been shown to be associated with accumulation of matrix representing the development of engineered cartilage (Kandel et al., 2020). In the case of NIR spectra of healthy cartilage with underlying subchondral bone, the ratio of water absorbances at 5,200 cm^−1^ and 7,000 cm^−1^ have been shown to reflect the increase in the ratio of thickness of subchondral bone to articular cartilage (Mcgoverin et al., 2014).

In addition to spectroscopy in the NIR region, analysis in the visible spectral region (Vis; 350–700 nm) can provide important information on tissue composition. This spectral region has been used extensively for characterization of the light interaction with different biological tissues, including breast tissue, forearm muscle, abdomen, and forehead. In these studies, absorbances from tissue components such as deoxygenated and oxygenated hemoglobin, lipids, and water were monitored to yield information related to composition and/or function (Taroni et al., 2003; Jacques, 2013). In an osteochondral defect, this spectral region may provide information on hemoglobin, which would indicate the presence of a blood clot and/or neovascularization in the repairing tissue, typically present in the early stages of repair and less so at the later stages.

The overall aim of this study was to evaluate the potential of Vis-NIR fiber optic spectroscopy to assess molecular changes related to tissue repair in osteochondral tissues *in situ* in a porcine preclinical model. This was carried out by collection of spectra from tissues at the defect site, followed by experiments to facilitate spectral interpretation. The depth of detection of diffuse reflectance spectra in the Vis-NIR spectral regions was also investigated.

## Methods

### Animal Study

Animal procedures were approved by the Institutional Animal Care and Use Committee (IACUC) at the University of Pennsylvania and performed in the animal facility at the University of Pennsylvania. Animals in the current study were part of larger studies at U Penn, where the mature and immature animals comprised separate studies. For the 1-month study, six skeletally mature (age 12 months at the beginning of the study) Yucatan minipigs (Sinclair Bioresources, Auxvasse, MO, United States) were used, while for the 3-month study, three juvenile (age 6 months at the beginning of the study) Yucatan minipigs (Sinclair Bioresources, Auxvasse, MO, United States) were used. In each animal, full thickness chondral defects were created bilaterally in the trochlear groove using a 5-mm biopsy punch, at proximal, middle, and distal locations of the medial and lateral sides (six defects in the right hind limb per animal, as part of a larger study). MFx treatments were randomized from joint to joint to enable consistency with regard to their load-bearing location. Awl-based microfracture procedures (diameter: 0.8 mm; depth: 2 mm) with three marrow stimulation holes were carried out. In total, there were *n* = 9 microfracture-treated cartilage defects in the 1-month animal study and *n* = 6 microfracture-treated cartilage defects in the 3-month animal study. The animals were housed and recovered under veterinary care. At the end of the study period (1 or 3 months post-surgery), all animals were euthanized, and the tissues were evaluated spectroscopically and histologically (in addition to other end-point tests related to the original studies, and not reported here).

### Vis-NIR Spectra Collection

Vis-NIR spectra were collected from the microfracture-treated defect tissue and from native healthy cartilage immediately after euthanasia *in situ* in intact joints. Spectra were collected in a diffuse reflectance configuration using a fiber optic reflectance probe (Malvern Panalytical, United States) with six illumination fibers (600 microns diameter) and one collection fiber attached with a 2-mm spacer on the tip of the probe. The probe was connected to an ASD LabSpec four Standard-Res Lab Analyzer spectrometer (Malvern Panalytical, United States). The fiber optic probe tip diameter was approximately 3 mm. As the osteochondral defects were created using a 5-mm-diameter biopsy punch, we consistently collected data from the center portion of the repair tissue. These spectral data reflected average absorbances over the 3 mm diameter region, which, based on the awl diameter of 0.8 mm, would include a combination of punctured and non-punctured subchondral bone regions. From each tissue region, *n* = 3 spectra were collected, with 50 co-added scans and 8.5 ms of integration time over a spectral range of 350–2,500 nm. The spectra were divided into the visible region (350–750 nm) and the NIR region (1000–2,500 nm or 10,000–4,000 cm^−1^). All the visible region spectra were presented as wavelength (nm), and the NIR region spectra were presented as frequency (wavenumber, cm^−1^) per convention. The spectra were processed in ViewSpec Pro (Malvern Panalytical) for splice correction of detector regions. The corrected spectra were then processed in Unscrambler X (CAMO, Norway) software. The spectra were smoothed using the Savitzky–Golay (SG) filter with nine smoothing points. Second-derivative processing was used (SG filter with 15 points smoothing), and then the spectra were inverted to evaluate the positive intensity of peaks of interest. Outcomes from spectral analysis in the NIR region were absorbance peak intensities reflecting water content (peaks at 7,000 and 5,200 cm^−1^) and matrix protein content (peaks at 5,900 and 5,800 cm^−1^), while absorbance peaks reflecting oxygenated hemoglobin (blood) content (peaks at 540 and 570 nm) were investigated in the visible region (van Kampen and Zijlstra, 1983).

### Vis-NIR Depth of Detection Into Joint Tissue

**FIGURE 1:**
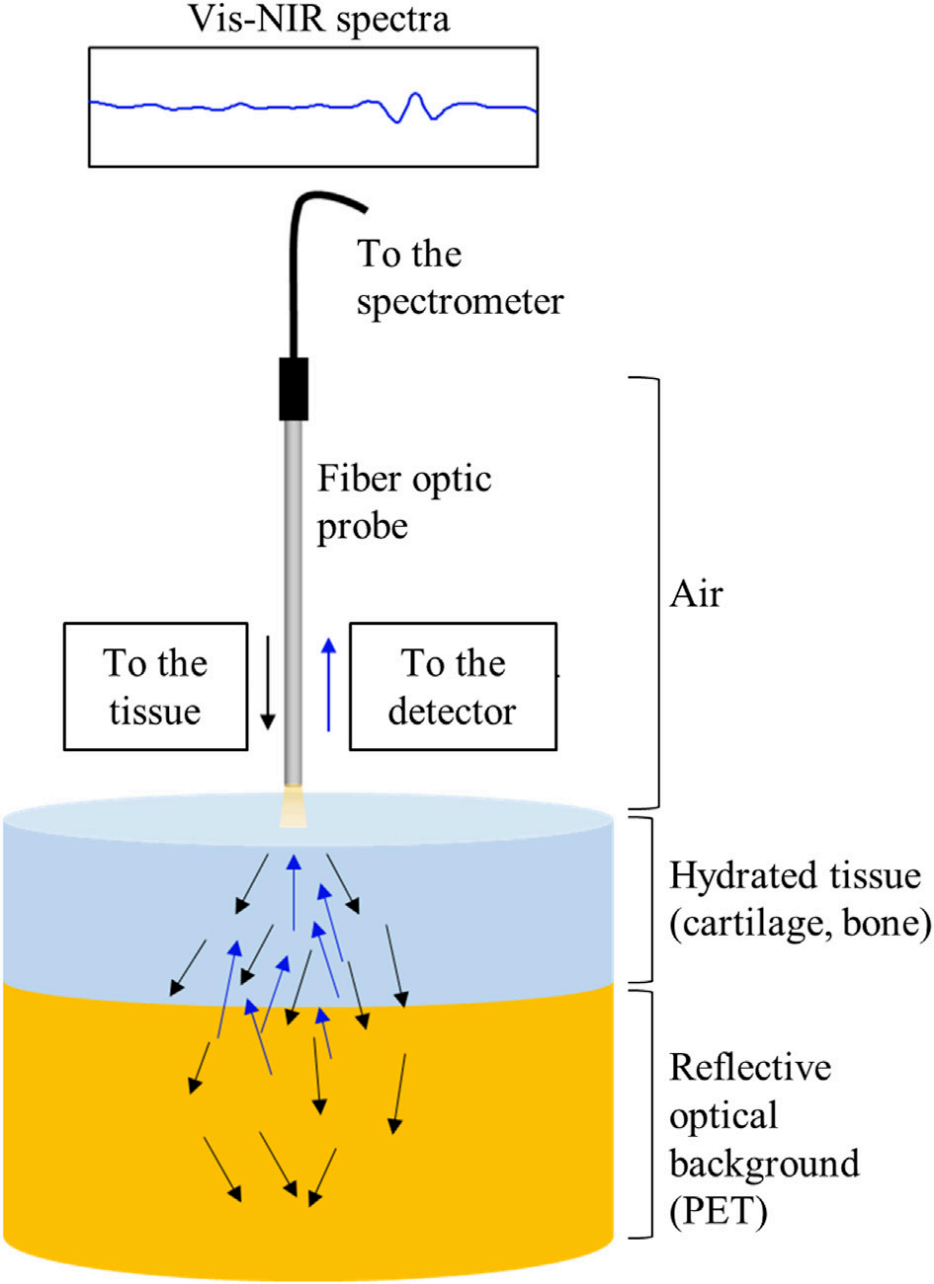
Schematic of light scattering through cartilage tissue and a semi-infinite reflecting surface.

The extent of light interaction from the fiber optic probe into the tissues was of interest to aid in spectral interpretation of the repairing joint tissue spectra. This was assessed as the depth of detection in the harvested intact joint tissue and in the cartilage and bone separately and together. Harvested repair tissue from the 1-month microfracture study (primarily fibrous tissue, based on histology) was also investigated separately. Briefly, fresh stifle joints from one adult Yorkshire pig (Animal Biotech Inc. Doylestown, PA) were dissected, and multiple full osteochondral plugs were harvested using a 6-mm biopsy punch (Miltex). Vis-NIR spectra of tissues of varying thicknesses of articular cartilage (1.6–4 mm), subchondral bone (1–3 mm), and intact total joint tissue (cartilage and bone, 3–6 mm) were collected on top of 25 mm of polyethylene terephthalate (PET). PET acts as a diffusely reflective optical background with semi-infinite optical thickness and also is characterized by unique spectral absorbances (Zonios and Dimou, 2006). (Figure 1). The PET polymer was chosen as it has distinct peaks in the spectral regions of interest. In the NIR region, the 8,865 cm^−1^ (1,130 nm) absorbance has been previously used to characterize PET polymer (Moroni et al., 2015) and arises from the 2nd overtone of C-H stretching; the PET absorbance at 6000 cm^−1^ (1,660 nm) arises from 1st overtone of aromatic C-H stretching plus methylene stretching (Lachenal, 2006). The ability to detect these absorbances while collecting spectral data from tissues on top of PET can establish the depth of detection. Similarly, in the visible region, PET has a characteristic peak at 375 nm (Ouchi et al., 1999). The depth of detection into the tissue was determined based on the minimum thickness of tissue from which the PET signal was not detected in the spectra, similar to that previously described in Padalkar and Pleshko, (2015). A Spectralon standard underneath the tissues was used as a control for data collection.

### Histology

Full depth explants of the defect repair tissue regions were fixed in 4% paraformaldehyde for 24 h. Samples were decalcified for 8 weeks by placing the samples in 50-ml conical tubes with 15–20 ml of decalcification solution (Formical-2000) with intermittent changes, followed by embedding in paraffin wax. To account for variations in subchondral histomorphology with sectioning depth into the tissue block, we obtained sections approximately from the center of each defect region, observing the size of the defect region as guide to select the appropriate sections (larger defect, more central defect position). The embedded samples were sectioned at 8 microns thickness onto the glass slides, and for each sample, the section with the largest defect region was stained with Safranin-O (0.2%) and fast green (0.02%) and imaged under a bright field microscope. One adjacent section from each block was obtained for Fourier transform infrared (FTIR) spectral imaging, as described in the following section.

### Histological ICRS II Scoring

Four blinded independent observers previously trained in ICRS scoring of tissues reviewed and scored control and defect sections using the ICRS II Histological Assessment Scale (Mainil-Varlet et al., 2010). While all parameters that comprise the ICRS II scale were scored on a scale from 0 (poor defect repair) to 100 (normal articular cartilage), only the following parameters having significant differences between the two groups were reported: overall average score, matrix staining, surface architecture, basal integration, subchondral bone abnormality, and vascularization. The four observer scores obtained for each parameter were averaged.

### FTIR Spectroscopic Imaging for the Assessment of Defect Thickness

As it was challenging to assess defect thickness based on the contrast in the histology sections, FTIR imaging data were collected from unstained sections of 5 microns thickness on low-e slides (Kevley Technologies). Sections were imaged using a Spotlight 400 imaging spectrometer (Perkin Elmer) with a spectral resolution of 8 cm^−1^, spatial resolution of 25 μm, and two co-added scans. Spectral images were analyzed using ISys 5.0 software (Malvern Instruments). The ratio of the integrated areas of the collagen absorbance centered at 1,338 cm^−1^ to the amide II protein absorbance (West et al., 2005) was used to define the contrast related to the defect area. For each defect, 10 measurements were taken and averaged to calculate the defect thickness in each sample.

### Data Analysis

Results of measurements were reported as means and standard deviations. Comparisons of parameters between 1- and 3-month repair tissues were assessed by a Wilcoxon Rank Sum Test (ICRS II scores ad sub-scores) or Student’s *t*-test (defect thickness and spectral parameters), considering *p* < 0.05 for significance.

## Results

### Depth of Vis-NIR Detection Into Cartilage Tissue

**FIGURE 2:**
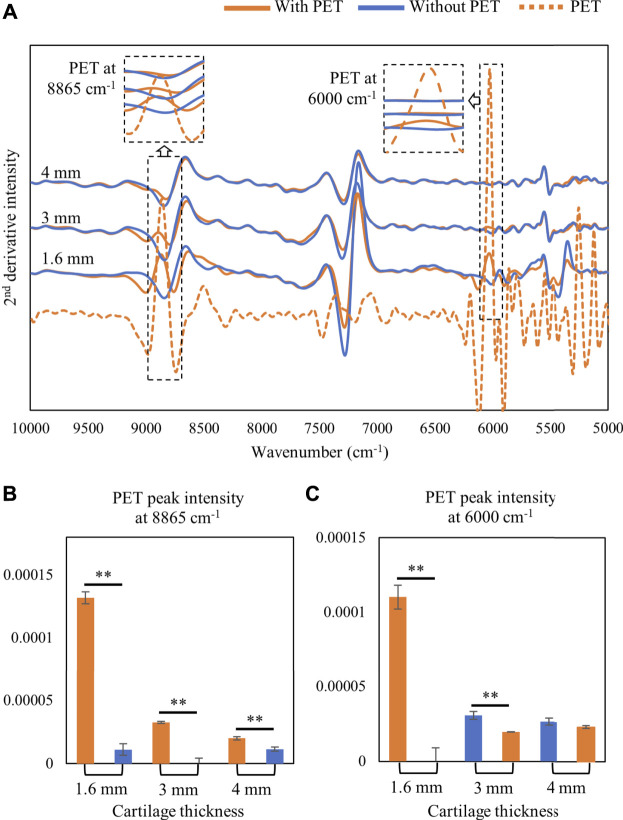
**(A)** Second-derivative spectra of harvested cartilage tissue collected on top of a semi-infinite PET block or on Spectralon standard (without PET). The depth of detection of PET signal at higher and lower frequencies was monitored by assessment of the PET signal at 8865 cm^−1^ and 6000 cm^−1^, respectively. **(B)** 8865 cm^−1^ signal intensity can be detected with a cartilage thickness of up to 4 mm. **(C)** 6000 cm^−1^ PET signal intensity can be detected with a cartilage thickness of up to 3 mm only. ∗∗*p* < 0.01.

The detection depth of the NIR signal into tissue was assessed by monitoring the signal intensity of PET signature peaks at 8,865 and 6,000 cm^−1^ (Figure 2A). The 8,865 cm^−1^ peak could be detected through 4 mm of cartilage thickness as shown in Figure 2B; however, in the lower frequency range, the 6000 cm^−1^ absorbance was only detected through 3 mm of cartilage (Figure 2C).

**FIGURE 3:**
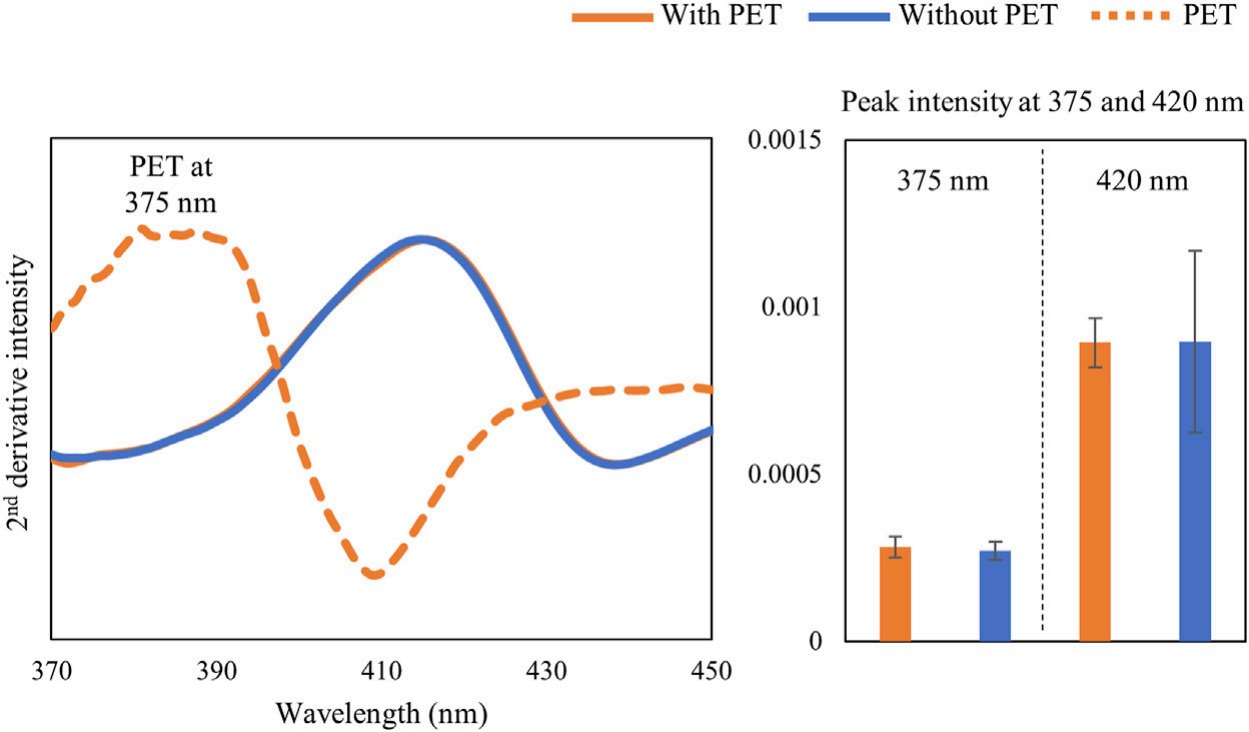
**(A)** Second-derivative spectra of cartilage tissue (1.6 mm) with and without a semi-infinite PET polymer (25 mm) and the polymer alone showing the PET peak at 375 nm and cartilage peak at 420 nm. **(B)** Second-derivative peak intensity with and without PET underneath cartilage tissue.

It is known that light penetration into biological tissue is greatly diminished in the visible region (Ash et al., 2017). Thus, it was not surprising that the PET signal at 375 nm was not detectable even through a cartilage thickness of 1.6 mm (Figure 3).

### Histological Scoring of Cartilage Tissue Repair

**FIGURE 4:**
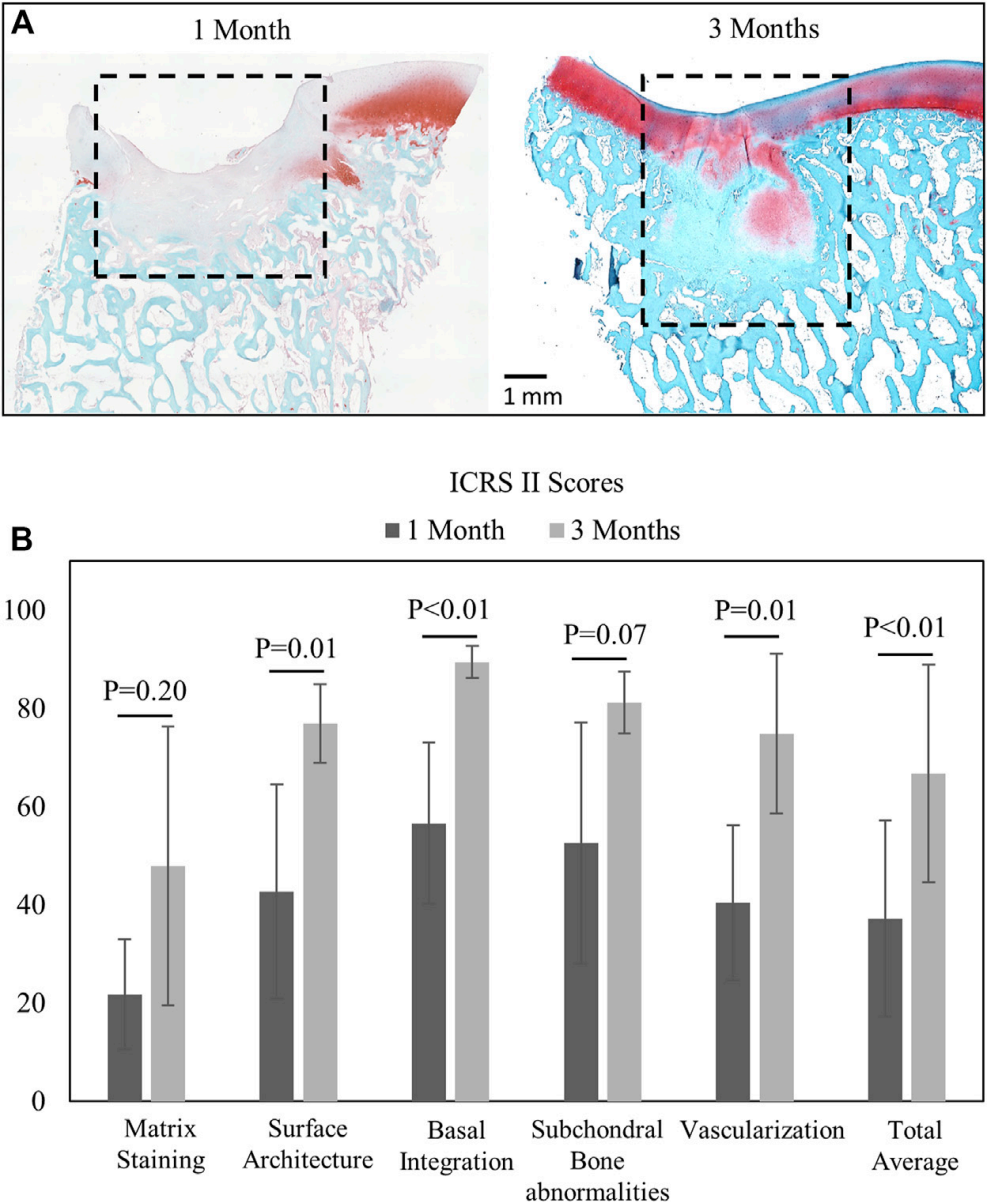
**(A)** Saf-O- and fast green-stained images from 1-month (*n* = 9) and 3-month (*n* = 6) animals with the defined repair tissue region. **(B)** Mean and standard deviation of ICRS II component scores (comparisons by the Wilcoxon rank sum test, significance at *p* < 05).

Histological staining and blinded ICRS II scoring revealed better repair after 3 months in the juvenile animals than 1 month of repair in the mature animals (Figures 4A,B). As expected, repair tissue at 3 months had higher combined total average score (Figure 4B) and better surface architecture, basal integration, and vascularization within the repair tissue than the 1-month repair tissues (*p* < 0.01).

### Defect Thickness

**FIGURE 5:**
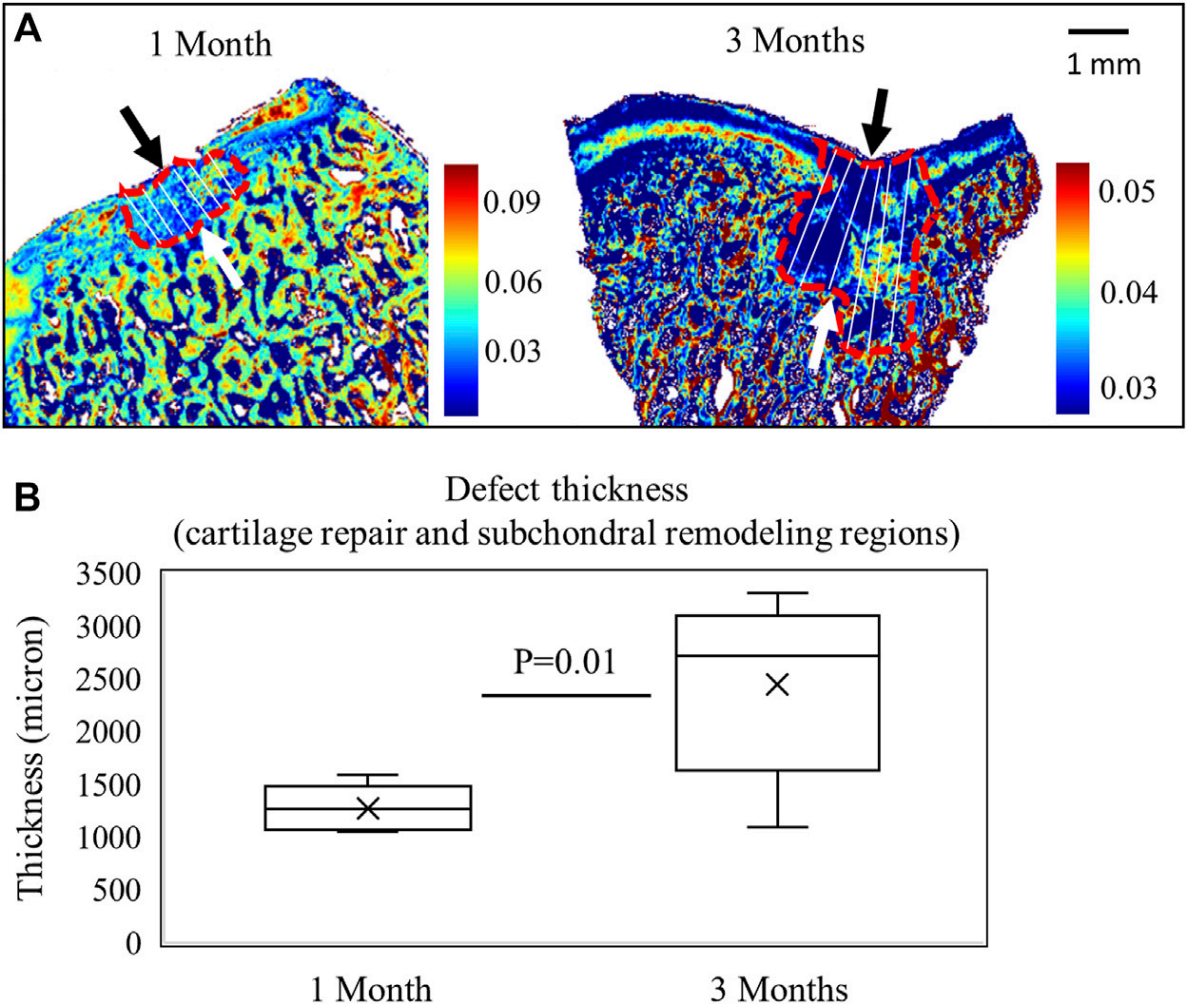
**(A)** FTIR images based on the 1338 cm^−1/^Amide II areas after 1 month of repair in a mature animal (*n* = 9) and 3 months of repair in a juvenile animal (*n* = 6). The measurement lines of defect thickness are shown, including both cartilage repair and subchondral remodeling regions. **(B)** Defect thickness is significantly greater in the 3-month animals (Student’s *t*-test).

Sample images after 1 and 3 months of repair are shown in Figure 5A. The defect thickness (comprising cartilage repair and subchondral remodeling regions) is significantly higher in 3-month juvenile animals than 1-month adult animals (*p* < 0.05) (Figure 5B). The increase in defect thickness seems to be primarily due to a more expansive subchondral remodeling seen in the juvenile animals, which is typical of juvenile osteochondral tissue healing following MFx (Pfeifer et al., 2017).

### Vis-NIR Spectroscopic Analysis

**FIGURE 6:**
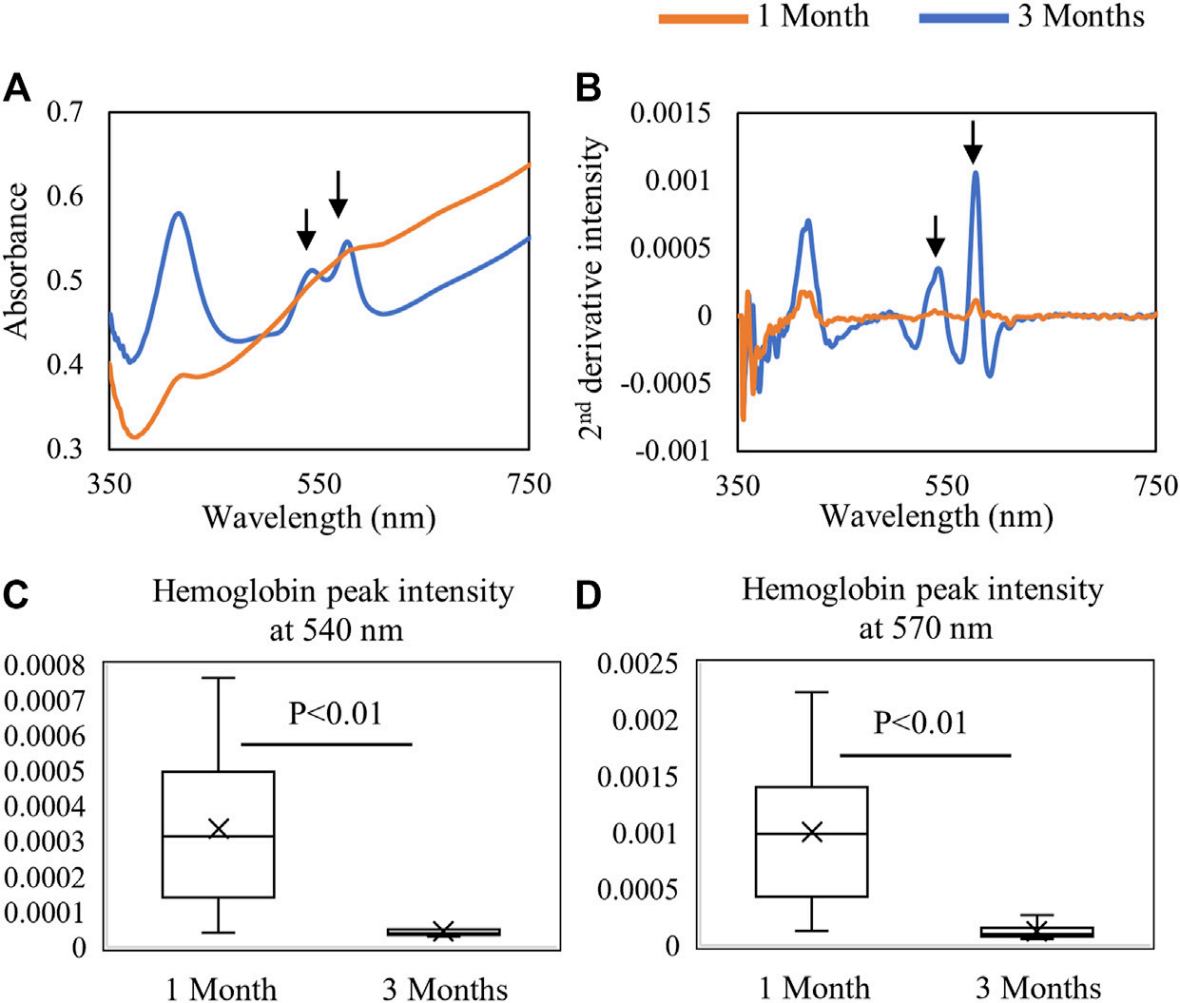
Average **(A)** raw and **(B)** second-derivative inverted Vis-NIR spectra from 1-month (*n* = 9) and 3-month (*n* = 6) defects *in situ*. The visible spectra clearly depict the differences in hemoglobin absorbance (540 and 570 nm) between 1-month and 3-month repair. Quantified second-derivative peak intensities of hemoglobin peaks at **(C)** 540 nm and **(D)** 570 nm showing greater hemoglobin detection in the 1-month tissue (Student’s *t*-test).

**FIGURE 7:**
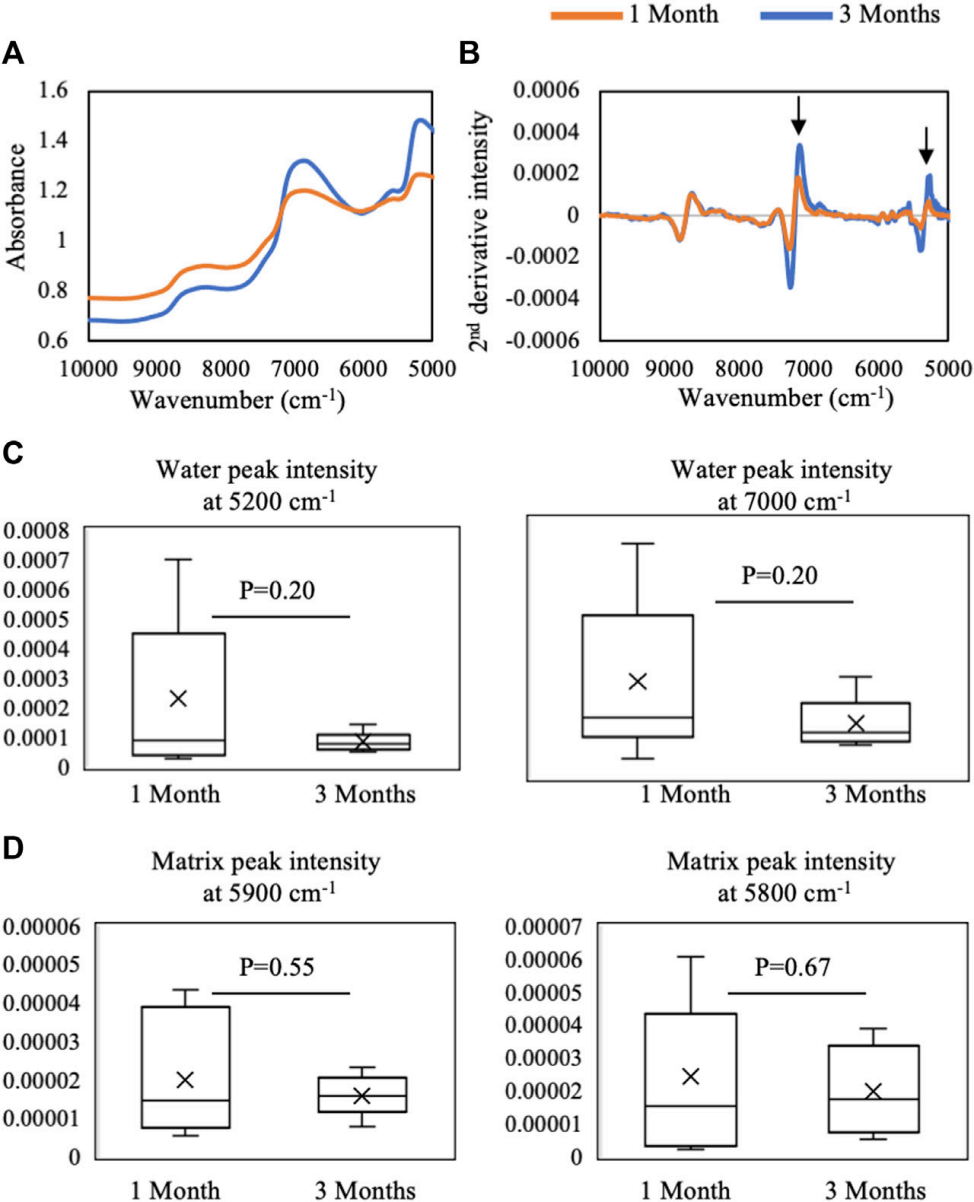
Average **(A)** raw and **(B)** second-derivative inverted NIR spectra from 1-month (*n* = 9) and 3-month (*n* = 6) defects *in situ*. The spectra highlight qualitative differences in water absorbances at 5,200 cm^−1^ and 7,000 cm^−1^ between 1 month and 3 months of repair. Quantified second-derivative peak intensities of **(C)** water absorbances and **(D)** matrix absorbances in 1- and 3-month defects. Although trends are present, no significant differences were found in intensities between 1- and 3-month defects (Student’s *t*-test).

Fiber optic-based Vis-NIR spectra were used to non-destructively assess the properties of the repair tissue at 1 and 3 months post-surgery. The spectra were divided into the visible region (350–750 nm) as shown in Figure 6 and the NIR region (10,000–5,000 cm^−1^) as shown in Figure 7. The raw spectra in the visible region showed differences between 1 and 3 months repair tissue based primarily on differences in hemoglobin absorbance peaks at 540 and 570 nm (Figure 6A). The inverted second-derivative spectra clearly showed the differences in hemoglobin absorbances (Figure 6B), which could be quantitatively assessed based on peak intensities (Figures 6C,D).

In the NIR region, the average raw spectra of 1-month repair tissue showed qualitatively more water absorbance and a slightly higher baseline offset than 3-month repair tissue (Figure 7A). The second-derivative processing revealed that differences between 1- and 3-month repair tissue were primarily in water absorbances at 7,000 and 5,200 cm^−1^ (Figure 7B), but quantification did not yield significant differences (Figure 7C). Similarly, quantification of NIR parameters reflecting protein content showed no significant differences (*p* > 0.05) (Figure 7D).

**FIGURE 8:**
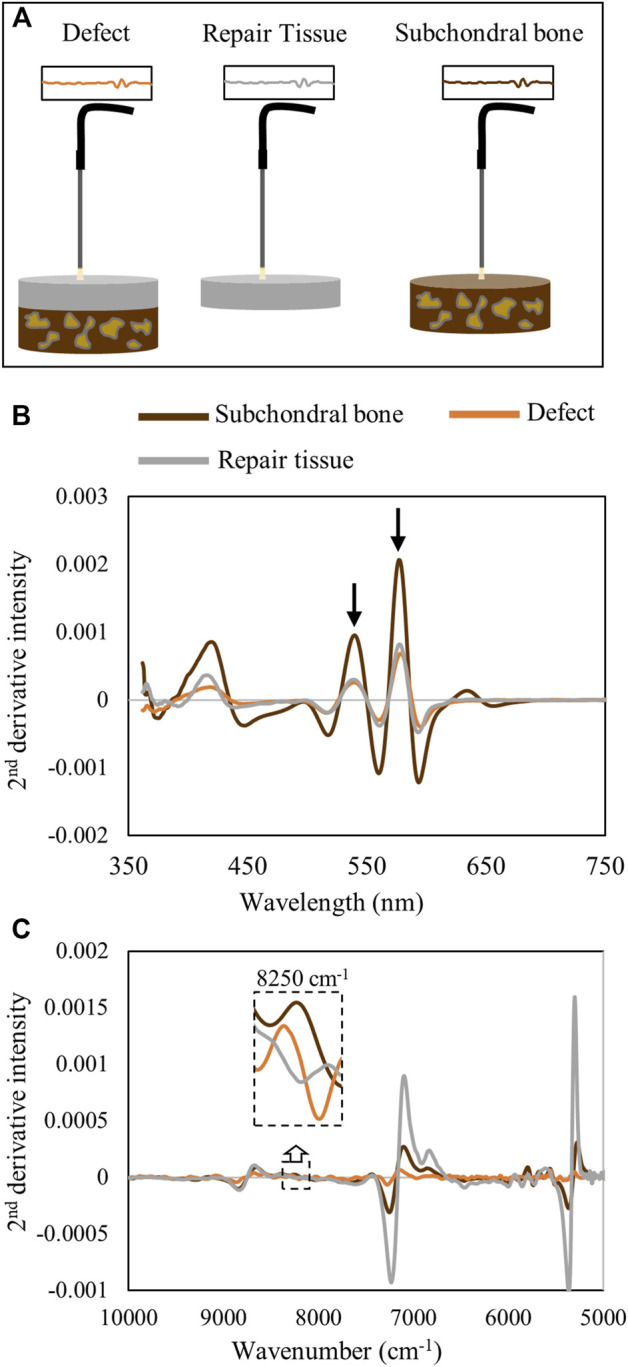
**(A)** Schematic of data collection from the defect tissue (*in situ*) and individual components, repair tissue, and subchondral bone, *ex vivo*. **(B)** Second-derivative spectra from the defect *in situ* and isolated repair tissue and subchondral bone *ex vivo* in the visible range demonstrate the hemoglobin peak intensities at 540 and 570 nm in the defect tissue are more similar to those in the repair tissue than to those in subchondral bone. **(C)** Second-derivative spectra in the NIR region demonstrate the differences in the water absorbances in the repair tissue in *ex vivo* compared to *in situ* spectra. At 8250 cm^−1^, the influence of subchondral bone on the *in situ* defect spectrum is apparent while absent in the isolated repair tissue spectrum.

To confirm that the hemoglobin peaks were primarily arising from the soft repair tissue, and not from underlying subchondral bone, spectra of isolated repair tissue were collected and compared with those from the entire defect *in situ*, as well as with those from isolated subchondral bone (Figure 8A). The averaged spectra show that the hemoglobin absorbances at 540 and 570 nm from the *in situ* defect are similar to the absorbance from the isolated repair tissue (Figure 8B). This is in line with our previous observation of the shallow depth of detection of visible light in this spectral region (Figure 3). In contrast, in the NIR region, the water absorbances are much higher in the isolated repair tissue *ex vivo* than in the defect *in situ*, and the subchondral bone spectrum more closely resembles the *in situ* defect spectrum (Figure 8C). The subchondral bone influence on the NIR spectra of the defect can also be observed in the 8250 cm^−1^ region, which shows the absorbance peak in both subchondral bone and in the defect, while the isolated repair tissue does not have this absorbance.

## Discussion

Cartilage defects and degeneration cause disability and pain in millions of people worldwide. Accurate evaluation of tissue pathology and progression of repairing cartilaginous tissues would aid in management of defect repair, potentially preventing progression to widespread tissue degeneration (Jones, 2009; Li et al., 2020). Arthroscopy is the gold standard for investigation of intra-articular pathology of the knee (Dehaven and Collins, 1975; Paatela et al., 2020). Conventionally, arthroscopic assessment of joint tissues is carried out to qualitatively assess the tissue using visual or tactile evaluation using a hook probe. Arthroscopic scoring systems such as Oswestry Arthroscopy Score (OAS) can be used to measure the quality of repair following articular cartilage surgery and can be used as a semiquantitative outcome measure (Smith et al., 2005; Paatela et al., 2020). However, the use of a tactile probe can be highly subjective and thus suboptimal (Brismar et al., 2002; Spahn et al., 2009). Vis-NIR spectroscopy using portable spectrometers and fiber optic probes offers a powerful alternative approach for the quantitative assessment of cartilage and subchondral tissue. Previously, NIR spectroscopy equipped with a fiber optic diffuse reflectance probe was used to investigate cartilage and subchondral bone properties in juvenile equines where information from the NIR first optical window (750–1,900 nm or ∼13,333–5263 cm^−1^) was found to be useful for characterizing and estimating subchondral bone properties, such as bone volume fraction, bone mineral density, and thickness and thus potentially could be adapted for arthroscopy (Sarin et al., 2018). However, those outcomes were based on artificial neural network analyses, which require very large sample sizes. Here, we highlight an expanded spectral range to include the visible region hemoglobin absorbances. This was shown to be a valuable tool for assessment of changes in *in vivo* repair tissue in a Yucatan minipig chondral defect model and potentially in other animal models and clinical scenarios.

Assessment of defects by conventional ICRS II histological scoring showed differences in many of the scored parameters between the 1- and 3-month timepoints. As expected, the repair tissue improved over time, even though there was significant bone remodeling observed at the 3-month timepoint. Such remodeling in this juvenile minipig cohort is unsurprising, as younger Yucatan minipigs undergo greater bony changes post-microfracture than adult animals (Pfeifer et al., 2017). Nonetheless, one of the most notable results from this study was the ability to detect the presence of varied amounts of blood in the repairing defects, which could be useful for longitudinal monitoring of the repair process. Although the NIR region of the *in situ* spectra did not show significant differences in spectra from 1- and 3-month repair tissue, some overall trends were apparent, which could likely be better defined with future multivariate spectral analyses, such as those carried out in other studies (Sarin et al., 2018; Prakash et al., 2019). Furthermore, it appeared that the NIR spectral features from the defect region were more similar to the subchondral bone than to the soft repair tissue. This is in line with the findings of Sarin et al. (2018), in which NIR spectral data from intact joints reflected subchondral bone properties.

The process of tissue repair in the joint environment is complex and can take months, or even years. Post-surgery defect repair occurs in three biological phases: the clot formation phase, repair cartilage formation phase, and cartilage maturation phase (Mithoefer et al., 2012). Immediately after microfracture is performed, the defect region is filled with a blood clot rich in mesenchymal stem cells from subchondral bone marrow (Steadman et al., 2010). This initial phase is also marked by increased neovascularization, with capillary blood vessels from the marrow invading the defect region (Shapiro et al., 1993). Along maturation, this blood-rich tissue is gradually replaced by cartilaginous repair tissue over time; thus, the reduction in blood content can be used as a spectral marker for the progression of cartilage repair.

The timeline for the clot phase and subsequent cartilaginous tissue repair varies depending on characteristics of the native tissue and defect. Shapiro et al. (1993), in their study of repair of full-thickness articular cartilage defects in rabbit models for over a period of 24 weeks, found that cartilage repair begins with fibrinous clot and invasion of blood vessels for few days followed by extracellular matrix synthesis as early as 10 days and fibrocartilaginous tissue with chondrocytes present by 3 weeks. From 6, 8, 10, and 12 weeks, progressive differentiation of cells into chondroblasts, chondrocytes, and osteoblasts and synthesis of cartilage and bone matrices in their appropriate locations was found with both tidemark and compact lamellar subchondral bone plate formation to occur by 24 weeks (Shapiro et al., 1993).

It is important to note, however, that this relatively quick repair progression may be related to the animal model, as rabbit cartilage has been described as having high potential for spontaneous healing (Chu et al., 2010). It is thus possible that in humans and large animals, the clot phase may persist for over weeks before progressive maturation into fibrocartilage repair tissue (Fortier et al., 2012; Guermazi et al., 2015). Here, our results indicate that the amount of blood clot and/or neovascularization in the repair tissue is greater in adult pigs after 1 month of defect creation than in juvenile animals after 3 months. The more intense neovascularization is observed by the lower ICRS II scoring (indicating more presence of blood vessels), and the greater blood content (possibly in the clot and/or within blood vessels) was detected by Vis-NIR fiber optic spectroscopy.

Understanding the resultant spectra collected from a defect comprised several tissue types is challenging due to overlapping absorbances from the multiple tissues present. During Vis-NIR spectroscopic data collection of cartilage or repair tissue, the incident light has variable penetration into the soft tissues (hyaline cartilage, fibrocartilage, and fibrous tissue) and the underlying subchondral bone. An earlier study by Padalkar and Pleshko, (2015) showed that using the diffuse reflectance fiber optic mode of spectra collection, NIR light can penetrate into cartilage tissue and detect more than 4 mm in 7,000–9,000 cm^−1^ and up to 3 mm in 5,000–7,000 cm^−1^. The study provided an approximation of light interaction at different thicknesses of articular cartilage. Thus, there is potential for the resultant NIR signals from joint tissue to arise from a combination of tissue types. However, it is also well known that measurement depth of the NIR diffuse reflectance depends in part on the geometry of the optical fiber probe used for illumination and detection of light from biological tissue. Arimoto et al. (2005) showed quantitatively that the measurement depth of NIR diffuse reflectance light into human skin depends on both the geometry (distance between illumination and detection fibers) and thickness of optical fibers in the probe. The determination of depth of detection by light into the defect is critical to evaluate the progression of repair in the defect. In our estimation, we have found that the repair tissue thickness increases from 1.5 mm in 1 month to ∼ 3.5 mm in 3 months (Figure 5), and that NIR light detection into the tissue ranges from 3 mm in low-frequency regions (6,000 cm^−1^) to more than 4 mm in high-frequency regions (8,865 cm^−1^) (Figure 2). Thus, the NIR light collected through the defect repair tissues interacts with multiple features and tissues of the defect area, assessing both soft tissue and subchondral bone repair regions. This may be a confounding factor contributing to why it was not possible to detect significant changes in NIR spectra between 1- and 3-month repair tissues. This is particularly evident in a higher frequency CH-overtone peak at 8,250 cm^−1^ (Burns and Ciurczak, 2007; Mcgoverin et al., 2014) which has a greater absorbance in subchondral bone and in the defect spectra than the soft repair tissue (Figure 8C). Mcgoverin et al. (2014) have previously observed that this peak absorbance is more related to subchondral bone than cartilage in osteochondral tissue. In our study, we also found the influence of subchondral bone on the *in situ* defect spectra based on this peak.

Interestingly, the intensities of the water absorbance peaks at 5,200 cm^−1^ and 7,000 cm^−1^ were not significantly different in the *in situ* defect spectra from 1- to 3-month repair tissues. This was surprising, as differences in these peak intensities reflect the relative amount of bone and cartilage, respectively, as the 5,200 cm^−1^ absorbance is typically greater than the 7,000 cm^−1^ in bone. This discrepancy is likely due to the influence of free water from the joint that is confounding the spectral comparisons between the two groups. Comparison to spectra from free water supports this possibility, as the 5,200/7,000 cm^−1^ ratio observed in the *in situ* data is more similar to that in free water (<1) (Padalkar et al., 2013) than to that in bone (>1.2) or repair tissue (>1.2) in the current study. This ratio has also been found to be a determinant of healthy cartilage where the ratio is lower than 1, while in degraded cartilage it is greater than 1 (Brown et al., 2011).

Diffuse reflectance spectra in the visible region have a lower depth of detection than that in the NIR spectral region. Ash et al. (2017) found that the interaction of light in the visible range of 400–600 nm is reduced in depth by nearly 5 times compared to that of the NIR range. Thus, in the visible range, the light interaction in the defect primarily occurs within the top soft tissue layer. Nevertheless, the visible region was very useful for assessment of changes in hemoglobin related to blood clot formation and remodeling with microfracture. Ideally, this would be implemented in collection of data from individual defects over the course of a study, which would also provide better information related to spectral changes associated with repair across all spectral regions. The lack of significant differences in NIR spectral parameters between the two groups studied here can also be attributed to the inherent individual variations in the tissue repair process, in particular at early timepoints. Kim et al. (2015) highlighted the importance of longer duration studies, 1 year to 2 years, to fully investigate the repair of the cartilage defects in Yucatan minipig models.

It is important to acknowledge limitations of this study with respect to comparing repair tissue from animals of different ages. This issue can be highlighted based on the study by Pfeifer et al. (2017), which described age-dependent differences in cartilage repair in juvenile (6 months) and skeletally mature (18 months) Yucatan minipigs. Similar to the animals in the current study, they evaluated tissue repair at the stifle joints after creating full thickness defects in the trochlear groove followed by treatment with MFx. After 6 weeks post-operatively, it was noted that the juvenile pigs had more extensive subchondral bone remodeling but overall better quality of repair tissue. Here, we analyzed tissue repair after 1 month using adult animals, whereas juvenile animals were used to analyze repair after 3 months. In line with results from Pfeifer et al., our juvenile animals had better quality repair tissue (ICRS II scores) but a more extensive subchondral bone remodeling (quantified here as a greater defect thickness). It is possible that these differences arise not only from the length of time of post-operative tissue repair but also as a difference between juvenile and adult animals. This would be an important confounding factor with respect to making conclusions about the progression of MFx-based cartilage repair.

Another confounding factor relates to differences in tissue sections analyzed for ICRS scoring and for thickness measurements by IR imaging. Several sections were cut for histology, and the section from the center-most region of the defect was chosen for ICRS scoring. As a result, sections for IR imaging analysis of tissue thickness came from off-center regions. This discrepancy leads to the potential for differences in the appearance of subchondral remodeling and healing within the same defect, which is reflected in Figures 4, 5. Figure 5 shows virtually no subchondral remodeling in the 1-month tissue, while some remodeling is apparent in Figure 4, taken from the center region of the defect. In spite of these confounding factors, the primary contribution of this study, assessment of defect repair by Vis-NIR spectroscopy, remains the same. Differences in composition between repair tissue with better (juvenile animals, 3 months of repair) or poorer (adult animals, 1 month of repair) quality can be investigated using this modality.

## Conclusion

Together, these data demonstrate a novel application of non-destructive fiber optic Vis-NIR spectral analysis for evaluation of chondral microfracture repair. Results from this study provide new insights into spectral parameters to assess the composition of cartilage repair tissue *in situ*, including hemoglobin absorbances, which may be a valuable approach toward quantitative assessment of tissue repair. Furthermore, with advancements in the development of arthroscopic fiber optic probes (Spahn et al., 2007; Sarin et al., 2018; Magnus et al., 2021), this study can have a significant impact as a foundation to inform on the design of future preclinical and clinical studies for a longitudinal spectroscopic analysis of chondral and osteochondral defect healing.

## Data Availability

The raw data supporting the conclusion of this article will be made available by the authors, without undue reservation.
